# Development of bread wheat (*Triticum aestivum* L) variety HD3411 following marker-assisted backcross breeding for drought tolerance 

**DOI:** 10.3389/fgene.2023.1046624

**Published:** 2023-02-24

**Authors:** Prashanth K. C. Kumar, Amasiddha Bellundagi, Hari Krishna, Mallana Gowdra Mallikarjuna, Ramya K. Thimmappa, Neha Rai, P. Shashikumara, Nivedita Sinha, Neelu Jain, Pradeep K. Singh, Gyanendra Pratap Singh, Kumble Vinod Prabhu

**Affiliations:** ^1^ ICAR-Indian Agricultural Research Institute, New Delhi, India; ^2^ ICAR-Indian Institute Millets Research, Hyderabad, India; ^3^ ICAR-Indian Institute of Oilseed Research, Hyderabad, India; ^4^ ICAR-Indian Grassland and Fodder Research Institute, Jhansi, India; ^5^ ICAR-Indian wheat and Barley Research Institute, Karnal, India; ^6^ PPV & FR Authority NAAS complex, New Delhi, India

**Keywords:** drought tolerance, wheat, MAS, foreground selection, background selection

## Abstract

Marker-assisted backcross breeding enables selective insertion of targeted traits into the genome to improve yield, quality, and stress resistance in wheat. In the current investigation, we transferred four drought tolerance quantitative trait loci (QTLs) controlling traits, *viz* canopy temperature, normalized difference vegetative index, chlorophyll content, and grain yield from the drought-tolerant donor line, C306, into a popular high-yielding, drought-sensitive variety, HD2733. Marker-assisted selection coupled with stringent phenotypic screening was used to advance each generation. This study resulted in 23 improved lines carrying combinations of four drought tolerance QTLs with a range of 85.35%–95.79% background recovery. The backcross-derived lines gave a higher yield under moisture-deficit stress conditions compared with the recipient parent. They also showed higher phenotypic mean values for physiological traits and stability characteristics of HD2733. A promising genotype, HD3411, derived from this cross was identified for release after national multi-location coordinating trials under the All India Coordinated Wheat Improvement Project. Our study is a prime example of the advantages of precision breeding using integrating markers and phenotypic selection to develop new cultivars with desirable traits like drought tolerance.

## Introduction

Wheat is a golden winter cereal grain and a major contributor to food and nutritional security, but the increase in drought due to climate can severely limit wheat production (Reynolds et al., 2001). Out of 12 distinct mega environments for wheat cultivation classified by the CIMMYT, only three are irrigated environments (Rajaram et al., 1995). The Indian sub-continent, which comes under the fourth mega environment, has a large area under wheat cultivation but only with residual soil moisture from the monsoon rains (Richards et al., 2001). In the last few years, global warming has turned researchers’ attention toward the drought tolerance of crop plants (Kang et al., 2009). It has been predicted that every 1°C increase in global warming leads to a decrease of 4.0%–6.5% in wheat production (Trnka et al., 2019). In the present context, threats of climate change and erratic behavior of climatic factors have raised awareness of the critical need to devise new ways to overcome drought stress.

Insufficient moisture affects wheat growth at phenological, reproductive, and grain-filling phases (Pradhan et al., 2012). Drought during the seedling stage will affect all the following growth stages and ultimately reduce grain yield (Sallam et al., 2022), and a prolonged mild drought during the flowering and grain-filling stages can cause 58%–92% reduction in grain yield (Matiu et al., 2017). Accurate field phenotyping for moisture stress adaption has been a critical issue in breeding for moisture stress tolerance (Mir et al., 2012; Tuberosa, 2012). At present, high-throughput phenotyping platforms are readily available for measuring phenotypic data. The normalized difference vegetative index (NDVI) and SPAD chlorophyll meter readings are related to plant health; higher values under moisture stress conditions are associated with greater vegetation and higher chlorophyll content. Such tools have been efficiently utilized in breeding for moisture stress tolerance in rice, maize, and wheat (Subash et al., 2011; Lu et al., 2011). The productivity of wheat under drought conditions is strongly associated with various physiological features such as leaf characteristics (low canopy temperature, staying green, chlorophyll content, early ground cover, NDVI, etc.), water use efficiency, and yield component traits (thousand-grain weight, spike length, grain number per spike, etc.). Additionally, a positive correlation between NDVI and chlorophyll content and grain yield under moisture stress has been reported in wheat (Harikrishna et al., 2016). Integration of physiological traits with grain yield component traits is necessary to improve wheat yield in moisture-stressed environments (Araus et al., 2008; Reynolds and Langridge, 2016). However, combining multicomponent drought tolerance-associated traits into a single cultivar using traditional breeding strategies is difficult in practice. The availability of markers for the genes underlying drought tolerance traits and marker-assisted backcross breeding (MABB) schemes can overcome the limitations associated with conventional introgression breeding.

Wheat breeding is rapidly changing, owing to advances in wheat genomics and molecular biology. The application of genomics technologies aims to realize faster and more efficient genetic gains of desirable traits. The discovery of RFLP marker technology led to the application of molecular markers in plant breeding (Tanksley et al., 1989), which are key tools for breeders in selecting desirable lines from germplasm and segregating generations (Raghavendra et al., 2020). The locking of genomic regions with the help of markers transformed the conventional backcross breeding into MABB, which is considered highly efficient in terms of time and cost and precision in selecting target traits. A plethora of QTLs and meta-QTLs for physiological and grain yield component traits have been reported by various studies (Quarrie et al., 2006; Kirigwi et al., 2007; Olivares-Villegas et al., 2007; Pinto et al., 2010; Kumar et al., 2012; Shukla et al., 2015; Gupta et al., 2017; Sunil et al., 2020; Puttamadanayaka et al., 2020; Khaled et al., 2022), which have enriched our knowledge of the genetic architecture of drought tolerance in wheat. The markers that have been validated, the stable QTLs, and meta-QTLs with more than 10% of explained phenotypic variance for drought tolerance traits have high practical utility in MABB to reconstruct drought-tolerant versions of important wheat cultivars. MABB is an accelerated approach for QTL introgression through marker-assisted foreground and background selection (Hospital and Charcosset, 1997). Marker-assisted foreground selection helps identify the gene of interest without extensive phenotypic assays (Tanksley, 1983; Melchinger, 1990), and marker-assisted background selection significantly expedites the rate of recovery of recurrent parent genomes with one or two backcrosses (Young and Tanksley, 1989; Visscher et al., 1996). MABB also produces a set of recombinant backcross inbred lines, which can be further evaluated to select the best recombinants from both parents (Manu et al., 2020; Shashikumara et al., 2020). So far, MABB has been exploited in all major crops: in rice, for bacterial blight resistance (Sundaram et al., 2008); in wheat, for powdery mildew (Zhou et al., 2003); and quality traits including high molecular weight (HMW) glutenins (De Bustos et al., 2001) and preharvest sprouting (Kumar et al., 2010). There are only a few reports on MABB application for drought tolerance in wheat, however (Rai et al., 2018; Gautam et al., 2020; Merchuk-Ovnat et al., 2016).

Keeping those mentioned previously in mind, we executed this study of the transfer of QTLs contributing to drought tolerance from the donor line C306 into the recipient variety HD2733 using the MABB approach. We successfully transferred QTLs for NDVI, chlorophyll content, low canopy temperature, and grain yield into the elite Indian wheat variety, HD2733. The improved lines with targeted QTLs were drought-resilient with higher grain yields under moisture stress conditions.

## Materials and methods

The experiment material consisted of the HD2733 cultivar, one of India’s most popular wheat varieties for the northeastern plain zone (NEPZ). However, HD2733 is susceptible to drought stress, resulting in substantial yield losses under field conditions of limited irrigation. The donor parent for the drought tolerance QTL was C306. This cultivar is suitable for drought-prone and rain-fed conditions and has been widely adopted in the NEPZ and NWPZ (northwestern plain zone).

### Parental lines and targeted QTL regions for transfer

We targeted four QTLs associated with NDVI, chlorophyll content, canopy temperature, and grain yield for marker-assisted introgression. The details of the targeted QTL and the flanking markers are given in Table 1.

**TABLE 1:** Molecular markers used for introgression into HD2733 through foreground selection.

QTL/trait	Primer	Chromosome	Sequence of the marker	Position (cM)	Annealing temperature (°C)	Reported *R* ^2^%	Observed *R* ^2^%	References
NDVI	*Xgdm* 93	2A	F: AAA​AGC​TGC​TGG​AGC​ATA​CA	170	61	20	18.2	Oliver et al., 2007
			R: GGA​GCA​TGG​CTA​CAT​CCT​TC					
Canopy temperature (CT)	*Xbarc68*-*Xbarc*101	3B	F: CGA​TGC​CAA​CAC​ACT​GAG​GT	33	55	35–40	28.3	Kumar et al., 2012
			R: AGC​CGC​ATG​AAG​AGA​TAG​GTA​GAG​A					
			F: GCT​CCT​CTC​ACG​ATC​ACG​CAA​AG					
			R: GCG​AGT​CGA​TCA​CAC​TAT​GAG​CCA​ATC					
Yield and chlorophyll content	*Xgwm304*	5A	F: AGG​AAA​CAG​AAA​TAT​CGC​GG	59	60	15	22.6	Pinto *et al.* (2010)
			R: AGG​ACT​GTG​GGG​AAT​GAA​TG					
Chlorophyll content (CHL)	*Xgwm301*	2D	F: GAG​GAG​TAA​GAC​ACA​TGC​CC	107	60	11.2	11.9	Pinto *et al.* (2010)
			R: GTG​GCT​GGA​GAT​TCA​GGT​TC					

### DNA isolation, PCR conditions, and parental polymorphism survey

Total genomic DNA was isolated by a micro-extraction protocol (Prabhu et al., 1998). A polymerase chain reaction (PCR) was performed in a 10 μL total reaction volume containing 2–3 μL (60–70 ng/μL) DNA, 2.0 μL 109 buffer with 25 mM MgCl_2_, 0.5 μL dNTPs (10 mM) (Bangalore Genei, Bangalore, Karnataka, India), 1.0 μL each forward and reverse SSR primers (20 mM) (Sigma Inc., St. Louis, MO, United States), 0.3 μL Taq polymerase (3 U/μL) (Bangalore Genei, Bangalore, Karnataka, India), and 5.2 μL distilled water (sterile). Amplification of the template DNA was performed according to the annealing conditions for the wmc, gwm, barc, cfa, and cfd series of SSR markers used (Roder et al., 1998; Pestsova et al., 2000; Gupta et al., 2002; Somers et al., 2004; Quarrie et al., 2005; Kumar et al., 2012). Amplified products were resolved on a 3.2% agarose gel (MetaPhor, Lonza, Rockland, ME, United States), along with a DNA ladder size standard (MBI, Fermentas), stained with 0.5 μg/mL ethidium bromide (Amresco, Solon, OH, United States), and documented with a gel documentation system (Bio-Rad, Hercules, CA, United States). The donor parent, C306, and recurrent parent, HD2733, were screened with 700 SSR markers, including markers associated with targeted traits.

### Marker-assisted backcross breeding (MABB)

**FIGURE 1:**
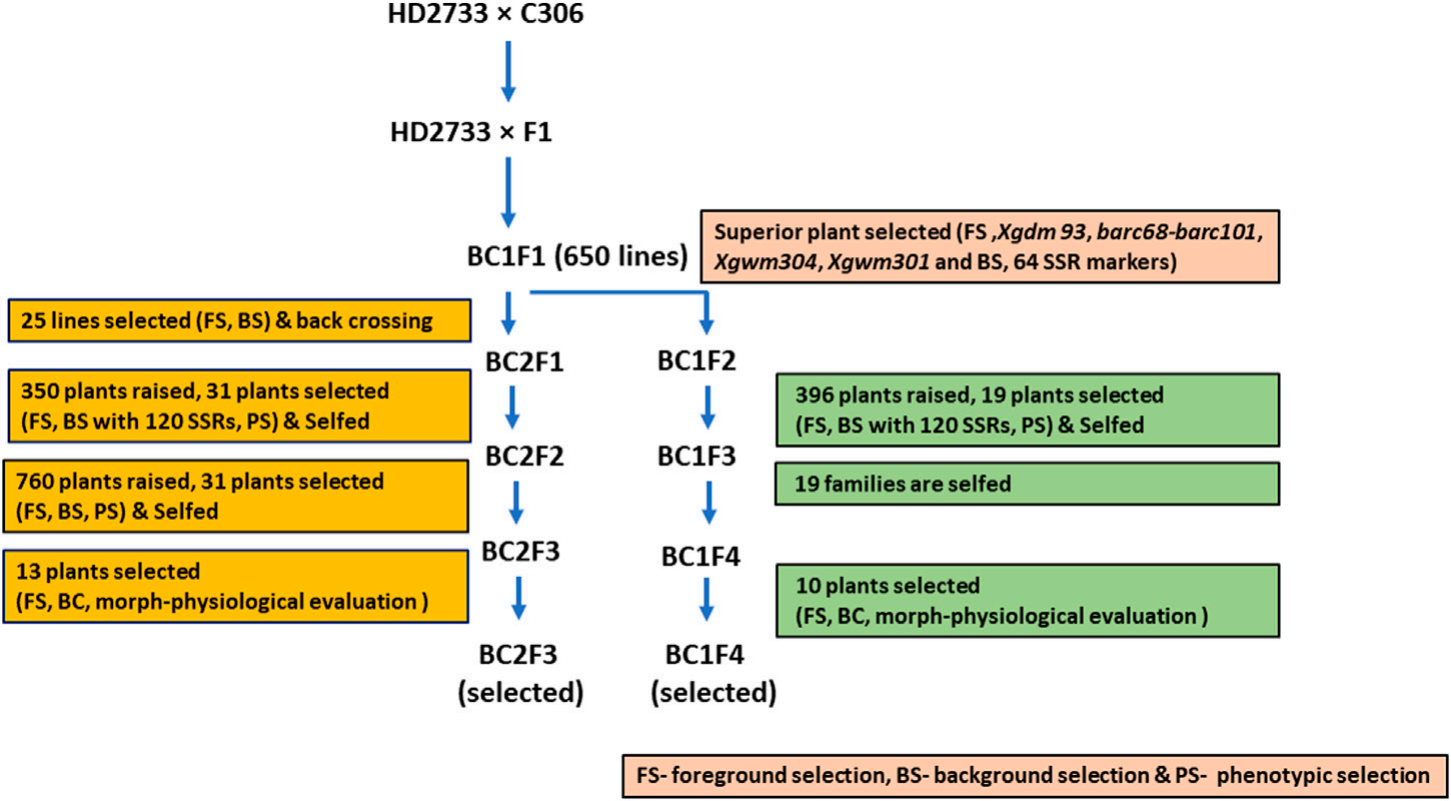
Schematic workflow of marker-assisted backcross breeding of HD2733 × C306.

Initially, a cross was made between HD2733 and C306 to transfer drought stress tolerance QTLs into HD2733. The MABB procedure followed here is represented in Figure 1. The true F_1_s were identified using foreground SSR markers and backcrossed to the recurrent parent. The BC_1_F_1_s were subjected to foreground and initial background selection with a set of 64 polymorphic markers. Twenty-five lines positive for target QTLs with maximum recurrent parent genome (RPG) recovery coupled with phenotypic similarity to the recipient parent were selected. The 21 selected lines were backcrossed to the recurrent parent and selfed to generate BC_2_F_1_ and BC_1_F_2_ seeds. BC_2_F_1_ and BC_1_F_2_ lines were repeated for the MABB process involving foreground and background selection with 120 polymorphic SSR markers.

The polymorphic SSR markers were used to construct a schematic map illustrating the genomic contributions of donor and recurrent parents with Graphical GenoType (GGT) v2.045 software to identify backcross-derived lines possessing the maximum recurrent parent genome. The positive foreground-selected plants genotyped for polymorphic markers at each backcross/selfing generation and recurrent parent genome recovery (G) were estimated using the following formula: *G* = [(*X* + ½*Y*) × 100]/*N*; here, *N* is the total number of parental polymorphic markers screened, *X* is the number of markers showing homozygosity for recurrent parental alleles, and *Y* is the number of markers showing heterozygosity for parental alleles. Based on the recovery of the recurrent parental genome and the presence of targeted donor genomic regions, 50 lines were selected from BC_2_F_1_ and BC_1_F_2_ plants and advanced through selfing.

Thirteen plants were selected from advanced BC_2_F_2_ lines based on maximum recovery for RPG through background and foreground selection and visible phenotypic similarity with the recurrent parent strain, HD2733, while BC_1_F_3_ lines were selfed and advanced to BC_1_F_4_ generations. A total of 10 BC_1_F_4_ plants were again selected based on foreground selection and maximum background recovery of the recurrent parental genome. The selected 13 BC_2_F_3_ and 10 BC_1_F_4_ plants were evaluated for morphological and physiological traits and yield performance and further advanced through selfing for evaluation under a national testing trial.

### Evaluation of morpho-physiological and yield component characteristics of the backcross-derived progenies for drought tolerance

The BC_1_F_4_ and BC_2_F_3_ families were raised under restricted irrigation conditions (two irrigations were carried out at 21 and 40 days after sowing), following an augmented design protocol, where parents were replicated as checks. The observations on introgressed progeny lines in the field for various traits contributing to drought tolerance and yield parameters were recorded as per CIMMYT guidelines published in “Physiological Breeding II: A field guide to wheat phenotyping” (Pask et al., 2012). The data were recorded for various morphological traits *viz*., 50% days to heading (DH), days to anthesis (DA), days to maturity (DM), plant height (PH), number of tillers (NT), spike length (SL), peduncle length (PL), 1,000 kernel weight (TKW), biomass, harvest index (HI), yield per plot (Y/P), and physiological traits like chlorophyll content, canopy temperature, and the normalized difference vegetation index were scored at three different stages of wheat development: the vegetative stage (late boot stage, Z49), the grain filling stage (early milk stage, Z73), and the grain maturity stage (late milk stage, Z85) according to Zadok’s scale (Zadoks et al., 1974).

## Results

### Marker–trait association of targeted drought tolerance QTLs

Foreground selection using the QTL-linked markers, *Xbarc68-Xbarc101, Xgdm93, Xgwm165,* and *Xgwm301*, associated with moisture stress tolerance were carried out on the MABB population. Initially, targeted QTLs were validated in the segregating BC_1_F_2_ population under restricted irrigation. Individual lines were phenotyped for associated traits and genotyped for targeted donor parent alleles. Single marker analysis was performed to arrive at the presence of QTL. It was observed that *Xgdm93*, linked to *qNDVI* located on the 2A chromosome, showed a phenotypic variance of 18.2% (*R* ^2^ = 0.182). *QChl.ksu-3B* and *QLt.ksu-3B*, flanked by markers *Xbarc68-Xbarc101*, showed phenotypic variance of 28.3% (*R* ^2^ = 0.283) for canopy temperature. The microsatellite marker, *Xgmw304* associated with QTL for grain yield and chlorophyll content on chromosome 5A, showed 22.6% phenotypic variation (*R* ^2^ = 0.226). Another QTL associated with chlorophyll content co-segregated with *Xgwm301* on chromosome 2D and depicted 11.9% phenotypic variation (*R* ^2^ = 0.119) in the segregating BC_1_F_2_ population (Table 1).

### Marker-assisted transfer of drought tolerance QTLs into HD2733

The crosses were made between HD2733 and C306 to improve drought tolerance in the recurrent parent HD2733, during *rabi* 2011. We simultaneously followed two approaches for the introgression of targeted QTLs: (1) where BC_1_F_1_ lines were allowed for the second backcross and advanced to generate BC_2_F_3_ and (2) where BC_1_F_1_ lines were selfed and advanced to the BC_1_F_4_ generation. The F_1_s obtained were confirmed by screening *XBarc68-Xbarc101, Xgdm93, Xgwm301, and Xgwm304* microsatellite markers polymorphic between HD2733 and C306. The true F_1_s were backcrossed with the recipient parent to produce 650 BC_1_F_1_s, which were confirmed for the presence of the QTL-linked markers, *Xgdm93, Xbarc68-Xbarc101, Xgwm304,* and *Xgwm301* (i.e., foreground selection). The foreground selection resulted in the identification of 79 lines (20 lines with *Xbarc68-Xbarc101+Xgdm93* and *Xgwm301+Xgwm304*; 18 lines with *Xgwm304+Xgdm93*; 17 lines with *Xbarc68-Xbarc101* + *Xgwm304*; 12 plants with *Xgwm304+Xgwm301*, and 12 lines with *Xgdm93+Xgwm301*) for background selection. The selected lines were examined for phenotypic differences for targeted traits (QTL expression) and phenotypic similarity with the recipient parent. These lines were also subjected to background selection with a set of 64 polymorphic SSR markers, and 25 lines possessing the maximum recovery percentage of the recurrent parent genome (74.5%–75.4%, Table 2) were identified. The selected lines were selfed and crossed to the recipient parent to generate a population of 396 BC_1_F_2_s and 350 BC_2_F_1_s plants.

**TABLE 2:** Range of genome recovery (%) in different backcross and selfed generations.

S. No	Generation	No. of selected plants	Recurrent parent genome (%)
1	BC_1_F_1_	25	74.5–75.4
2	BC_2_F_1_	31	86.60–92.34
3	BC_2_F_2_	31	85.73–94.87
4	BC_2_F_3_	13	92.06–95.79
5	BC_1_F_2_	19	78.84–81.35
6	BC_1_F_4_	10	85.35–88.34

**FIGURE 2:**
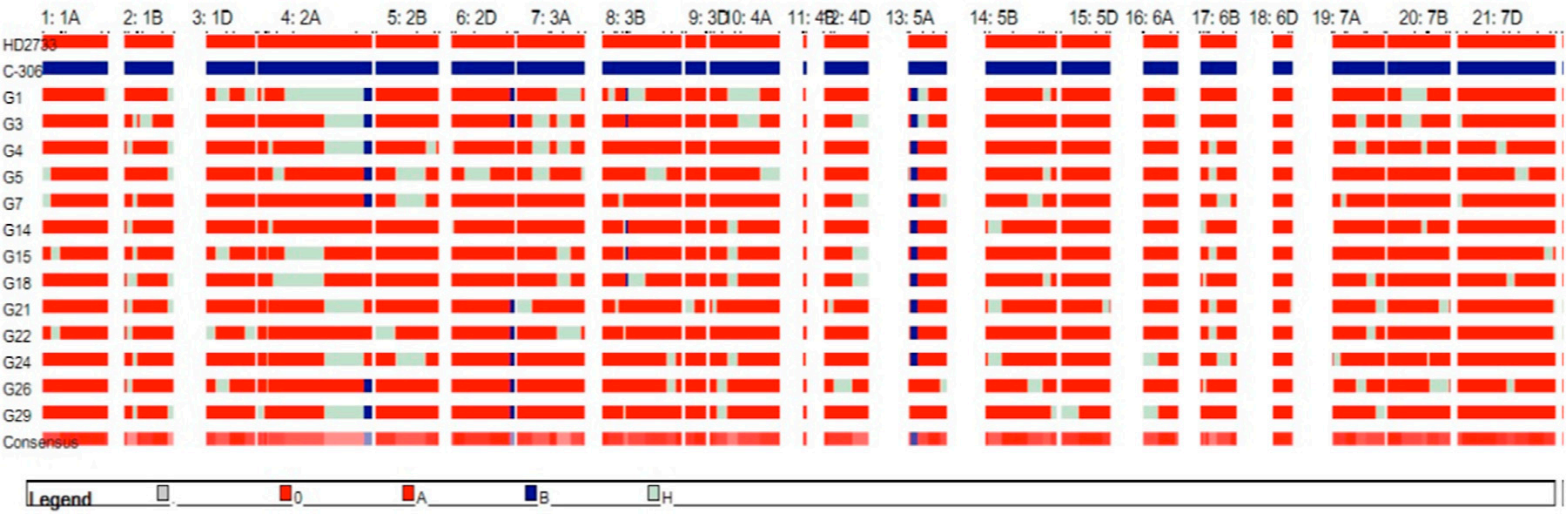
Genetic constitution of selected 13 BC_2_F_3_ lines with targeted QTLs on chromosomes.

For the 350 BC_2_F_1_s plants, the foreground protocol as explained previously was repeated (11 lines positive for *Xbarc68-Xbarc101+Xgdm93+Xgwm301+Xgwm304*, 18 lines positive for *Xgwm304+Xgdm93*, 17 lines positive for *Xbarc68-Xbarc101+Xgwm304*, 18 lines positive for *Xgwm304+Xgwm301*, and 18 lines positive for *Xgdm93+Xgwm301*), and a total of 82 lines with different combinations of introgressed QTLs were selected for background screening. Applying background selection using 120 polymorphic markers and considering the phenotypic similarity of lines with the recurrent parent, 31 BC_2_F_1_ lines were selected with an RPG recovery of 86.60%–92.34% (Table 2). In BC_2_F_1_, among a total of 31 selected plants, six plants possessed four QTLs (*qCT + qNDVI + qCHL + qYield*); eight plants carried two QTLs (*qYield + qNDVI*); six plants carried two QTLs (*qYield + qCT*); four plants carried two QTLs (*qNDVI + qCHL*); and seven plants represented two QTLs (*qYield + qCHL*). The selected plants were advanced to generate 760 segregating progenies in the BC_2_F_2_ generation. Among the 760 progenies, 31 lines homozygous for two or more donor parent marker alleles with a maximum RPG of 85.73%–94.87% were advanced to the BC_2_F_3_ generation. After reconfirmation for targeted QTLs and lines ranging from 90.90% to 95.79%, the RPG recovery was evaluated under drought stress conditions, where the crop was irrigated only once at a critical stage, i.e., 21 days after sowing (Table 2). Based on the performance of lines under drought stress for different morpho-physiological traits, 13 BC_2_F_3_ lines with RPG ranging from 92.06% to 95.79% were selected (Table 3). The graphical genotype of selected 13 BC_2_F_3_ lines shows that the distribution of donor parents’ genome segments was mostly restricted to targeted regions with an average of 88.27% of the recurrent parent genome (Figure 2).

**TABLE 3:** Percentage contribution of recurrent and donor parent alleles in selected MABB lines.

S. No.	Selected progeny	HD2733 allele %	Hetero. allele %	C-306 allele %	Total RPG %
BC_2_F_3_ lines					
1	HD2733-68-70-359	84.17	15.83	7.9	92.08
2	HD2733-33-59-141	89.17	10.83	5.4	94.58
3	HD2733-33-68-298	87.50	12.50	6.3	93.46
4	HD2733-571-296-642	89.17	10.83	5.4	94.39
5	HD2733-571-296-645	91.67	08.33	4.2	95.79
6	HD2733-33-64-203	89.17	10.83	5.4	94.86
7	HD2733-33-64-215	89.17	10.83	5.4	94.39
8	HD2733-365-262-622	88.33	11.67	5.8	94.86
9	HD2733-87-251-481	84.17	15.83	7.9	92.06
10	HD2733-571-296-657	89.17	10.83	5.4	94.39
11	HD2733-87-251-475	88.33	11.67	5.8	94.39
12	HD2733-361-37-97	88.33	11.67	5.8	93.46
13	HD2733-18-218-446	89.17	10.83	5.4	94.39
BC_1_F_4_ lines					
1	HD2733-20-1	73.33	26.7	13.3	86.67
2	HD2733-34-4	74.17	25.8	12.9	87.08
3	HD2733-34-5	75.00	25.0	12.5	87.50
4	HD2733-52-8	76.67	23.3	11.7	88.33
5	HD2733-56-9	72.50	27.5	13.8	86.25
6	HD2733-59-10	71.67	28.3	14.2	85.83
7	HD2733-62-11	70.83	29.2	14.6	85.42
8	HD2733-67-15	73.33	26.7	13.3	86.67
9	HD2733-70-17	75.83	24.2	12.2	87.92
10	HD2733-17-18	71.67	28.3	14.2	85.83

Note: C-306 allele % does not include the introgressed QTL regions of 2A, 2D, 3B, and 5A chromosomes.

**FIGURE 3:**
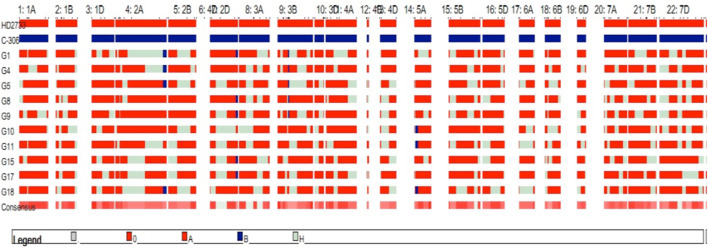
Genetic constitution of selected 10 BC_1_F_4_ lines with targeted QTLs on chromosomes.

Simultaneously, 396 BC_1_F_2_ lines were screened under foreground selection and targeted traits, including resemblance with the recurrent parent. A total of 37 BC_1_F_2_ plants, six with three QTLs (*qCT + qNDVI + qCHL*), eight with two QTLs (*qCT + qNDVI*), eight with two QTLs (*qCT + qCHL*), eight with one QTL (*qCHL*), and seven with one QTL (*qYield*) were selected for background screening using 120 polymorphic SSR markers. Of these, 19 BC_1_F_2_ plants with RPGs ranging from 78.84% to 81.35% were forwarded to the BC_1_F_3_ generation, and these 19 BC_1_F_3_ families were advanced to produce BC_1_F_4_ families. After confirming the presence of targeted QTLs with 85.35%–88.34% background recovery, the selected lines were examined under drought stress conditions (one irrigation at 21 days after sowing). Based on different morpho-physiological traits associated with the lines’ performance, 10 BC_1_F_4_ lines were selected for further evaluation. Graphical genotyping of the 10 selected BC_1_F_4_ lines revealed an average genome recovery of 73.50% from the recipient parent (Figure 3). Among all the transferred lines, the best included line in BC_2_F_3_ (HD2733-33-59-141, 4QTLs) with an RPG recovery of 94.58% and the one in BC_1_F_4_ (HD2733-20-1, 3QTLs) with an RPG recovery of 86.67% (Table 3).

### Evaluation of the improved lines of BC_2_F_3_ and BC_1_F_4_ for morpho-physiological traits

The improved lines containing QTLs in combination (four QTLs, two QTLs, and one QTL), derived from crossing HD2733 × C306, were evaluated in the augmented design along with parents as checks. The 13 BC_2_F_3_ and 10 BC_1_F_4_ lines selected were evaluated under drought stress for 26 different morpho-physiological traits and distinctiveness, uniformity, and stability (DUS) traits. DUS characterization was based on the plant’s ear shape and density, glume pubescence, growth attitude, foliage color, peduncle attitude, sheath-blade waxiness, and ear peduncle waxiness. We observed significantly higher phenotypic performance than HD2733 and a clear difference between lines for the trait-targeted MABB lines introgressed with respective QTL combinations. The details of the differences in traits are presented in Table 4 and Table 5. In particular, the three improved lines were pyramided with different combination of QTLs (*qCT + qCHL*, *qCHL + qYield*, and *qCT + qNDVI + qCHL + qYield*) in BC_2_F_3_, and BC_1_F_4_ showed a lower canopy temperature ranging from 18.3°C to 19.27°C compared to the recurrent parent, HD2733, with a canopy temperature of 21.0°C at the late boot stage. With respect to NDVI values, the improved lines were on par with the donor parent, C306, at the LB, EM, and LM stages. The average chlorophyll content of HD2733 at the LM stage under drought conditions was 37.40. We observed an improvement of up to 50.84 in selected BC_1_F_4_ lines with introgression of *qYield* QTL. Overall, the introgressed lines have shown significant improvement over the recurrent parents for lower canopy temperature, NDVI, stomatal conductance, and chlorophyll content (Table 5). The mean grain yield values were significantly higher (54.9-81.4 g) in BC_2_F_3_ and (55.4-74.0 g) in BC_1_F_4_ lines compared to the recipient parent HD2733with an average grain yield of 53.68 g (Table 4). Overall, based on superior phenotyping performance, DUS scoring, and a high percentage of background recovery, a total of 13 progeny lines in BC_2_F_3_ and 10 progeny lines in BC_1_F_4_ were selected.

**TABLE 4:** Mean performance of parents and selected MABB-derived lines with combinations of introgressed QTLs for phenological and agronomic traits.

Trait	HD2733 (rainfed)	C306 (rainfed)	BC_2_F_3_ lines					BC_1_F_4_ lines					
			*qCT + qNDVI + qCHL + qYield*	*qNDVI + qYield*	*qCT + qYield*	*qCHL + qYield*	*qCHL + qNDVI*	*qCT + qCHL + qNDVI*	*qCT + qNDVI*	*qCT + qCHL*	*qYield*	*qCHL*	*qYield + qNDVI*
DFL	80	71	80	78	78	79	81	73.67	74	76.33	75.75	75	76.5
DH	87	85	89	88	87	88	88	86.67	86	86.67	87.5	86.25	87
DA	90	88	92	91	91	91	91	88.33	88.67	89.67	91	88.75	91.5
DM	122	126	126	126	126	128	125	120.67	121	125.33	123.75	123.75	122.5
PH	78	97	78.3	80.1	77.1	83.2	77.1	77.53	71.93	78.83	77.88	84.63	90.75
SL	9.5	9.2	11.6	11.2	10.7	11.2	9.5	11.17	10.77	11.17	10.25	10.55	13
PL	54.5	44	39.7	40.4	37.6	44.8	39.6	37.3	39.27	36.83	38.5	38.13	39.15
SNPS	18.2	15.8	19.7	18.5	18.8	19.6	19	18.93	18.87	18.27	18.75	18.55	18
TNP	12.4	9.8	14.7	13.6	14.8	13.2	11.3	10	9.27	12.07	10.7	10.25	8.9
KNPS	200	177.00	221.70	213.70	225.90	244.8	221.1	203.33	236.67	196.67	222.25	238.5	210
TKW	46.9	45.45	45.9	45.5	46.4	42.3	44.7	38.57	38.39	43.79	42.31	46.77	42.98
HI	36.84	38.11	37.9	35.4	40.5	46.3	33.8	23.81	27.42	34.32	39.63	35.3	26.66
GY	53.68	60.97	79.3	78.1	80.1	81.4	65.3	58.46	55.37	69.58	64.48	74.03	54.95

DFL, days to flag leaf emergence; DH, days to heading; DA, days to anthesis; DM, days to maturity; PH, plant height; SL, spike length; PL, peduncle length; SNPS, spikelet number per spike; TNP, tillers per plant; KNPS, kernel number per five spikes; TKW, thousand kernel weight; HI, harvest index; GY, grain yield per five plants (grams); qCT, QTL related to canopy temperature; qNDVI, QTL related to the normalized difference vegetation index; qCHL, QTL related to chlorophyll content; qYield, QTL related to yield

**TABLE 5:** Mean performance of parents and selected MABB-derived lines with combinations of introgressed QTLs for physiological traits.

Trait	Stage	HD2733 (rainfed)	C306 (rainfed)	BC_2_F_3_ lines					BC_1_F_4_ lines						
				*qCT + qNDVI + qCHL + qYield*	*qNDVI + qYield*	*qCT + qYield*	*qCHL + qYield*	*qCHL + qNDVI*	*qCT + qCHL + qNDVI*	*qCT + qNDVI*	*qCT + qCHL*	*qYield*	*qCHL*	*qYield + qNDVI*	*CD @ 5%*
CT	LB	21.0	19.1	19.27	19.67	19.47	19.26	21.47	20.23	19.7	18.3	20.78	19.88	21.25	0.452
	EM	22.4	24.3	21.03	21.23	21.51	21.12	21.74	21	21.28	19.4	20.8	20.64	21.23	0.409
	LM	30.6	34.1	31.60	30.12	29.91	30.10	30.35	31.02	29.15	29.1	29.85	29.28	30.98	0.434
NDVI	LB	0.78	0.79	0.76	0.71	0.73	0.74	0.70	0.79	0.8	0.79	0.77	0.78	0.81	0.038
	EM	0.70	0.71	0.68	0.70	0.70	0.70	0.67	0.71	0.71	0.74	0.73	0.72	0.69	0.019
	LM	0.40	0.54	0.37	0.48	0.46	0.50	0.43	0.44	0.51	0.55	0.53	0.51	0.44	0.016
%GC	Seedling	46.16	47.68	25.68	18.07	19.16	14.56	16.47	20.01	20.2	20.52	21.37	19.5	17.95	1.012
Stomatal conductance	LM	337.4	359.2	477.53	377.63	462.29	407.56	387.00	438.4	584.8	416.6	371.23	445.25	474.2	8.521
	EM	500.7	364.3	478.89	504.76	481.48	516.07	470.10	508.89	583.56	406.33	335.25	545.58	481.67	20.24
Chlorophyll content	LB	41.42	47.12	49.72	49.65	48.84	49.74	49.25	50.94	45.38	47.76	48.06	49.32	49.3	0.314
	EM	50.40	50.54	52.76	51.89	53.09	52.49	51.99	53.43	50.98	50.24	52.9	54.36	53.38	0.521
	LM	37.40	42.08	28.97	39.61	46.38	43.91	37.39	40.23	47.96	38.86	50.84	47.89	41.14	1.112

CT, canopy temperature; NDVI, normalized difference vegetation index, %GC, percent ground cover; LB, late boot; EM, early milk; LM, late milk.

### Evaluation of HD3411 under the All India Coordinated Research Project (AICRP) on wheat and barley

Among the superior lines selected from BC_1_F_4_, one line, HD3411 with three major QTLs, has been submitted for a coordinated multi-location varietal trial under the All India Coordinated Research Project (AICRP) on wheat and barley. HD3411 performed better than its recurrent parent HD2733 and reported yield superiority for 2 years over check varieties DBW 39, DBW 187, and HD 3086 by 4.81%, 0.02%, and 5.94%, respectively, in national multi-location trials. HD3411 had a potential yield of 65.8 q/ha with an average yield of 46.75 q/ha under timely sown, irrigated conditions (Table 6). This variety also revealed a high level of resistance against leaf rust and moderate resistance to leaf blight, powdery mildew, kernel bunt, and flag smut. Based on yield superiority to its recurrent parent, this variety has been identified as suitable for timely sown, irrigated conditions in the northeastern plain zone (NEPZ) of the wheat growing region in India (AICRP on wheat and barley).

**TABLE 6:** Summarized yield data (q/ha) of All India wheat-coordinated varietal trials of HD3411.

	Year of testing	No. of trials	Proposed variety (HD 3411)			Check variety				Recurrent parent	C.D @ 5%
				HD3249	DBW187		DBW39	HD 2967	HD 3086	HD 2733	
Mean yield q/ha	2020–21	14	46.3	46.4	45.5		44.5	46.8	42.6	46.1	1.0
	2021–22	5	48.00	48.0	50.2		0.0	48.5	47.8	45.9	1.4
	Weighted Mean		46.75	46.8	46.74		44.5	47.2	44.0	46.0	
% Increase over the check cultivars	2020–21	14	-	−0.22	1.73		3.89	−1.08	7.99	0.43	-
	2021–22	5	-	0.00	−4.58		-	−1.04	0.42	4.38	-
	Weighted Mean			−0.16	0.02		4.81	−1.07	5.94	1.50	

CD, critical difference; locations, 2020–21 (14): Kanpur, Prayagraj, Ghaghra Ghat, Ayodhya, Gorakhpur, Sabour, Pusa Bihar, Cooch Behar, Kalyani, Burdwan, Manikchak, Ranchi, Chianki, and Dumka; locations, 2021–22 (5): Araol, Ayodhya, Gorakhpur, Burdwan, and Chianki

## Discussion

Drought stress alone causes greater yield loss in wheat than biotic stresses. Here, we have shown how the introgression of QTLs associated with water use efficiency performed through MABB can deliver improved stress-resilient products. The timely expression of introgressed QTLs in improved lines enhances water use efficiency and grain yield. Roots are the main organs that sense early moisture stress and try to compensate by extracting moisture from lower soil layers. Cooler canopy temperatures result in better evapotranspiration, which is an indirect measure of efficient water uptake by the roots from deep soil layers. Higher grain yield, chlorophyll content, and NDVI are key indicators of better photosynthetic efficiency of plants under moisture stress. So, we successfully infiltrated these key characteristics into the popular, but drought susceptible variety, HD2733.

The introgression of such complex traits with low heritability and unpredictable genotype × environment interactions is possible due to mapped QTLs associated with drought tolerance traits. MAS-based breeding is simple, efficient, robust, and accurate compared to conventional breeding methods that are time-consuming, laborious, and influenced by the environment. Numerous studies have reported QTLs for grain yield and component traits under drought stress conditions (Pinto et al., 2010; Gupta et al., 2012,, 2017; Puttamadanayaka et al., 2020). However, seldomly noted QTLs have been applied to improve drought tolerance in wheat (Merchuk-Ovnat et al., 2016; Rai et al., 2018; Gautam et al., 2020). In the present study, four drought tolerance QTLs (NDVI, *Xgdm 93*; canopy temperature, *Xbarc68-Xbarc101*; yield and chlorophyll content, *Xgwm304*; and chlorophyll content, *Xgwm301*, have been successfully introgressed into HD2733 using MABB along with combined phenotypic selection. Acuna-Galindo et al. (2015) reported major meta-QTL regions for drought and heat tolerance based on genomic regions identified by independent studies. Our three targeted QTLs were located in putative meta-QTL regions. MQTL26 co-localized with QTL *Xbarc68-Xbarc101* on chromosome 3B and MQTL38 co-localized with QTL *Xgwm304* on chromosome 5A. Another putative MQTL on chromosome 2D co-localized with *Xgwm301*. We transferred these three meta-QTL regions into the HD2733 background. Many drought tolerance component traits are associated with MQTL regions and are believed to be carrying genes underlying drought tolerance mechanisms. Therefore, such regions need to be fine-mapped and validated. The improved lines exhibited relatively higher grain yield under restricted irrigation/rain-fed conditions. The use of improved HD2733 in wheat breeding programs could disperse these QTLs into the backgrounds of genotypes derived from it.

### Marker-assisted transfer of drought tolerance QTLs

The most effective way to carry out introgression is by following MABB with stringent phenotypic selection for QTL expression and recurrent parent phenology (Collard and Mackill, 2008). In executing MABB, we considered the important factors mentioned by Frisch and Melchinger (2005), like the number of targeted gene/QTLs to be transferred, the marker map, the applied selection strategy, and the crossing scheme for efficient conversion of the recurrent parent. The reported markers need to be validated before executing MAS (Xu and Crouch, 2008; Nicholas et al., 2008). Therefore, initially in the BC_1_F_2_ population, we validated the presence of introgressed QTLs in wheat chromosomes 2A, 2D, 3B, and 5A that explained phenotypic variance from 11.9% to 28.3%. In the present investigation, foreground selection with two backcrosses (BC_1_F_1_ and BC_2_F_1_) efficiently introgressed four targeted QTLs in a combination of 2–4 QTLs per improved line. The effectiveness of foreground selection was confirmed by the improvement in grain yield of 47.7% in the four QTL-combined backcross-derived lines, compared with HD2733. Similarly, a 48.7% yield increase was observed in two QTL combinations with a QTL for grain yield. Furthermore, those lines carrying chlorophyll and NDVI QTLs produced 21.6% more grain than the recurrent parents, and the significant phenotypic QTL expression in the introgressed lines indicated very low background effects of the recipient genome. Drought tolerance is a complex phenomenon, governed by the combined effect of several QTLs. The plant has to undergo modifications from roots to leaves to meet the altered evapotranspiration demands of moisture stress. Therefore, the breeder has to insert several traits to improve water use efficiency. In wheat, a significant positive association between grain yield, NDVI, chlorophyll content, canopy temperature depression, and thousand kernel weight has been successfully established by Lopes and Reynolds (2012), Harikrishna et al. (2016), and Ramya et al. (2016). Hence, the strategic coupling of NDVI, chlorophyll content, and canopy temperature with grain yield had a complementary effect on productivity under drought-stress conditions.

**FIGURE 4:**
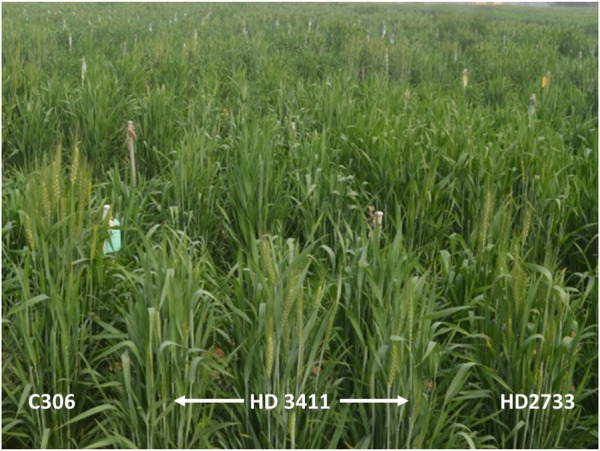
Field view of the variety HD 3411 with its parent ().

Prigge et al. (2009) showed that increasing marker density from early to advanced backcross generations resulted in maximum genome recovery with a minimum number of marker data points. Overall, 120 polymorphic SSR markers were sufficient to replace the recurrent parent genome in MABB. Additional backcrosses have produced benefits in increased background recovery to BC_2_F_3_ compared to BC_1_F_4_ (Table 3). Our targeted QTLs were dispersed over various chromosomes (2A, 3B, 5A, and 5D), which increased the chance of background recovery in respective chromosomes. Supporting markers assisted background selection by phenotypic selection for critical traits, which maximized the recipient parent genome reconstitution. Bhawar et al. (2011) reported 94.55% genome recovery in selected individuals of the BC_2_F_2_ generation in their study on pyramiding leaf rust-resistant genes into an elite cultivar, HD2687. Similarly, one MABB line, HD2733-571-296-645 from the BC_2_F_3_ generation had 95.79% background recovery. Fusion of gametes with donor and recurrent heterotic allelic combinations in BC1F1 gives rises to increased homozygosity in the progenies. Since, the majority of gametes in BC1F1 were segregating, random fusion might lead to combinations containing recurrent parent genomes, which could lead to enhanced recovery of RPG at the cost of residual heterozygosity. An MABB study by Chukwu et al., 2020 reported a recurrent parent genome recovery of 80%–86.4% from BC_2_F_1_ to 93.2%–98.7% from BC_2_F_2_, after one generation of selfing. Similar reports were presented by Bellundagi et al. (2022) for wheat and Miah et al. (2015) in rice. The potential application of background selection in accelerating the recurrent parent genome was thoroughly studied and discussed widely by many researchers (Servin and Hospital, 2002; Bai et al., 2006; Bhawar et al., 2011; Singh et al., 2012). The HD3411, the improved version of HD2733, derived from this cross has shown yield superiority over the recurrent parent under timely sown, irrigated conditions. A field view of HD 3411 with donor and recipient parents is given in Figure 4. Since this variety has been introgressed with QTLs for drought tolerance traits, it is also recommended for restricted irrigation conditions (AICRP on wheat and barley). We conclude that the success of MABB in delivering a drought-tolerant version of HD2733 is attributed to efficient foreground selection for different targeted QTLs, the combined effect of QTLs on yield in recurrent parent backgrounds, the screening of a large segregating population, and the presence of complementing markers that assisted background selection by phenotypic selection.

## Conclusion

The improved lines with different QTL combinations showed higher phenotypic mean values for respective traits (NDVI, chlorophyll content, low canopy temperature, and grain yield) compared to the recurrent parent. The backcross-derived lines carrying QTLs for both yield and physiological traits were superior in yield to the lines carrying QTL for either of the physiological traits alone. The four QTLs introgressed through MABB led to the development of drought-tolerant HD2733. Phenological traits such as days to flag leaf emergence, days to heading, days to anthesis, and days to maturity of back cross-derived lines were equal to the recurrent HD 2733 parent and slightly higher than the recurrent parent for yield-contributing traits. A total of 13 progeny lines in BC_2_F_3_ and 10 progeny lines in BC_1_F_4_ generations were found promising in performance under drought stress. One superior line HD3411 has shown higher yield over selected cultivars ranging from 0.02% to 5.94% after 2 years of a multi-location trial at the national level. The variety, HD3411, has been identified for varietal release and testing in the northeastern plain zone of the wheat-growing region in India.

## Data Availability

The raw data supporting the conclusion of this article will be made available by the authors, without undue reservation.
